# Low-Complexity User Selection for Rate Maximization in MIMO Broadcast Channels with Downlink Beamforming

**DOI:** 10.1155/2014/865905

**Published:** 2014-01-16

**Authors:** Eduardo Castañeda, Adão Silva, Ramiro Samano-Robles, Atílio Gameiro

**Affiliations:** ^1^Department of Electronics, Telecommunications and Informatics, Aveiro University, Aveiro, Portugal; ^2^Instituto de Telecomunicações, Campus Universitário de Santiago, P-3810-193 Aveiro, Portugal

## Abstract

We present in this work a low-complexity algorithm to solve the sum rate maximization problem in multiuser MIMO broadcast channels with downlink beamforming. Our approach decouples the user selection problem from the resource allocation problem and its main goal is to create a set of quasiorthogonal users. The proposed algorithm exploits physical metrics of the wireless channels that can be easily computed in such a way that a null space projection power can be approximated efficiently. Based on the derived metrics we present a mathematical model that describes the dynamics of the user selection process which renders the user selection problem into an integer linear program. Numerical results show that our approach is highly efficient to form groups of quasiorthogonal users when compared to previously proposed algorithms in the literature. Our user selection algorithm achieves a large portion of the optimum user selection sum rate (90%) for a moderate number of active users.

## 1. Introduction

Multiple-input multiple-output (MIMO) systems have a huge potential to attain high throughput in wireless systems [1, 2]. MIMO systems can be employed to exploit space-time coding and spatial multiplexing. When channel state information (CSI) is known at the transmitter, the overall system throughput can be increased by beamforming transmission. In the wireless scenario of interest, a transmitter encodes different information for different receivers in a common signal, which is referred to the literature as a broadcast channel (BC). For a classic deployment with one base station (BS) equipped with *N*_*t*_ antennas and *K* single antenna users, the overall throughput for a MIMO system increases by a factor of min{*N*_*t*_, *K*} the capacity of a time-division-multiple-access (TDMA) scheduling system if the transmitted signals are uncorrelated [1]. The TDMA system cannot exploit the multiple antenna deployment at the BS which leads to a waste of system resources and a limited system performance. The natural solution to this problem is to transmit simultaneously to more than one user. A strategy to accomplish this goal is to implement a nonlinear coding scheme called dirty paper (DPC) which is a multiplexing technique based on coding known interference [3]. The DPC exploits the full CSIT (at the transmitter) achieving the same capacity of an interference free MIMO BC system [2] and when the number of single antenna users *K* is larger than *N*_*t*_ at the BS, DPC can achieve a linear capacity increase in *N*_*t*_.

DPC is the optimal throughput maximization scheme in a MIMO BC system. However, it requires huge computation complexity and feedback information, which rapidly increases with *N*_*t*_. Two reduced-complexity suboptimal solutions to the throughput maximization problem were proposed in [4]. The first solution is the channel inversion zero-forcing beamforming (ZFBF) which is an orthogonal transmit spatial multiplexing linear precoding scheme whose main objective is to nullify the mutual interference among users according to perfect CSIT. Despite its simplicity, ZFBF has been shown to achieve the same asymptotic sum capacity of DPC when high multiuser diversity is ensured. The second solution called zero-forcing dirty-paper (ZFDP) is an asymptotically optimal beamforming scheme that combines a QR decomposition of the channel matrix with DPC at the transmitter. In this ranked known interference scheme, the first user is not affected by interference while the second user is only affected by interference coming from the first user. This procedure is repeated for subsequent users.

The throughput maximization using ZFBF (e.g., [4–8]) or ZFDP (e.g., [4, 9, 10]) can be further improved in scenarios where the number of single antenna users is larger than the number of antennas at the BS (*K* > *N*_*t*_). The users can be seen as an extra dimension of adaptation which is referred to in the literature as multiuser diversity. In order to exploit such diversity, it is necessary to select a set of active users whose channel characteristics result in a performance improvement (e.g., throughput) when they transmit simultaneously in the same radio resource. The user selection (scheduling) is a medium access control (MAC) process that can use information from the adaptive physical-layer (PHY) design so that temporal dimension (scheduling) and spatial dimension (multiple antennas) can be fully exploited. The scheduling is a real time process whose computational complexity and implementation efficiency affect directly the performance of upper-layers. Moreover, finding the set of users that optimizes a given global utility function is a highly complex combinatorial problem whose optimal solution is given by an exhaustive search and its associated search space grows geometrically with the number of users. Since the computation of the optimal solution to the scheduling problem is prohibited for most practical systems for moderate *K* and *N*_*t*_, it is necessary to find efficient suboptimal scheduling schemes that can provide a good trade-off between performance and complexity.

## 2. Related Works and Contributions

A considerable amount of work focused on the asymptotic sum rate of MIMO BC systems with user selection has been done over the last ten years (e.g., [5, 6, 8, 9]) and several published works presented efficient suboptimal algorithms that attempt to overcome the prohibitively high complexity of exhaustively searching users. Most of the works that suboptimally solve the problem of sum rate maximization in multiuser multiple-antenna systems implement cross-layer designs, where the scheduling decisions are made based on instantaneous CSI or link-level metrics.

Since the aforementioned problem can be tackled in different ways, we propose a classification of the algorithms that can be found in the literature based on the methodology followed to solve the mixed convex and combinatorial problem of throughput maximization in multiuser MIMO BC systems. We use this classification to make a clear distinction between the metrics used by each class and to fairly compare the performance achieved by algorithms of different classes.

We say that a class-A algorithm is the one that performs a joint user selection and power allocation optimization. A new user *k* is added to the set of selected users *𝒮* only if for a given utility function *U* the aggregation of *k* to *𝒮* increases the value of the utility function; that is, *U*(*𝒮*) < *U*(*𝒮* + {*k*}). This kind of greedy algorithms [10–15] are highly effective for throughput maximization. However, they still employ a high computational power since the selection process requires the evaluation of the global utility function (this requires a water-filling power allocation evaluation and the computation of the Shannon capacity) for each unselected user in every iteration of the algorithm.

The algorithm class-B operates in two phases. In the first phase a set of users is selected based on specific channel characteristics and in the second phase the algorithm evaluates the global utility function for the previously defined set [5, 6, 8, 9, 16]. This means that the user selection and the resource allocation (powers and beamforming weights) problems are carried out independently and the throughput maximization heavily depends on the channel characteristics of the selected users. Furthermore, the cardinality of the set of selected users is fixed in the first phase and it might be modified during the second phase when the global utility function is evaluated. For instance, if water-filling based power allocation is performed to evaluate the global utility function, this might result in zero power allocation for some selected users due to the channel characteristics of the selected users, the power constraints, and the SNR regime. In [5] the authors designed a greedy algorithm that performs a semiorthogonal user selection (SUS) in order to maximize the total sum rate implementing ZFBF. In this class-B algorithm the new selected user maximizes the component of the channel that is orthogonal to the subspace spanned by the channels of the previously selected users. The evaluation of that orthogonal component requires the multiplication of the unselected channel vectors by a matrix that describes the subspace defined by channels of the selected users. The authors of [5] showed that the average sum rate of ZFBF combined with their proposed user selection technique achieves asymptotically the average sum rate of DPC when the number of users is infinite (*K* → *∞*). Tu and Blum [9] proposed a class-B greedy algorithm for throughput maximization and ZFDP. The metric for user selection is based on the channel component projected onto the null space of the space spanned by the previously selected user channels. This metric is used to estimate the power degradation that a new user will experience if it interacts with the orthogonal subspace spanned by the other selected users. A statistical analysis of this methodology was done in [10], where it was shown that the greedy user selection based on channel component projection is a suboptimal yet highly efficient way to form groups of quasiorthogonal users that suboptimally maximize the sum rate. The main drawbacks of this approach are the following: one is the computation of a null space projector matrix unsing the channels of all selected users, and two is the multiplication of such projector matrix by the channels of all unselected users in order to identify the best unselected user. A similar approach to [9] was presented in [8] for throughput maximization with ZFBF. The difference between these two approaches lies in the fact that the latter performs singular value decomposition (SVD) in order to evaluate null space of the selected user channels. The user selection of [8] requires for each iteration the multiplication of the matrix that defines the null space of the selected channels by all nonselected channels.

### 2.1. Contributions

Both classes of algorithms require extensive use of matrix operations to perform the user selection. Class-A algorithms use matrix inversion in order to perform power allocation per each possible set of selected users and class-B algorithms require the computation of either the projector or the orthogonal projector matrix [17] per iteration and a matrix inversion for the final power allocation based on water-filling.

In this work we design a low-complexity suboptimal greedy class-B algorithm for throughput maximization that makes scheduling decisions based on simple physical metrics of the channels, that is, information extracted from the channel norms and the orthogonality between channels. We propose a metric that approximates the one used in [8, 9] with the advantage that we only require multiplication of scalars defined by the correlation coefficient between any two channels. We quantitatively compare the MIMO BC system performance in terms of the throughput (measured by the average sum rate) achieved by the proposed algorithm and several state-of-the-art algorithms (classes A and B).

The nature of the quasiorthogonal user grouping yields the maximization of the sum projection power of the selected users. The optimum sum projection power can be approximated as the optimization of a global objective function, which is given by the sum of individual weighted convex functions. For this problem the constraints are given by affine functions and the weights are given by binary variables. Therefore, we show that it is possible to render the sum projection power problem into a convex integer program which can be efficiently solved using available numerical packages. In contrast to previous works (e.g., [13]) that only provide a description of the user selection problem as an integer program (due to the high complexity of the problem formulation), we provide a complete mathematical model for the integer constrained program based on the derived metric whose solution asymptotically approximates the optimum one for moderate values of *K*.

Numerical results show that our proposed algorithms can achieve a large portion of the optimum sum rate with a low-computational complexity price and high performance for both precoding schemes ZFBF and ZFDP. Moreover, the proposed algorithms outperform state-of-the-art class-B algorithms for low values of *K* and achieve asymptotically optimal behavior for large values of *K*.

### 2.2. Organization

The remainder of the paper is organized as follows. In Section 3 we present the system model.  Section 4 describes the throughput maximization and the user selection problems and the optimization metric that is studied along the paper.  Section 5 presents the design of a greedy algorithm that performs quasiorthogonal user selection and a general mathematical model that represents the user selection problem as an integer programming problem.  Section 6 shows numerical examples for the assessment of the proposed algorithms using different performance metrics. The main conclusions are drawn in Section 7.

Some notational conventions are as follows. Matrices and vectors are set in boldface. 〈·〉, (·)^*T*^, (·)^*H*^, |·|, ||·||_*F*_, and *𝔼*{·} denote the inner product, transpose, hermitian transpose, set cardinality, Frobenius norm, and the expectation operation, respectively. Sp(**A**) denotes the subspace spanned by the rows of matrix **A**, rank(**A**) is the rank of matrix **A**, and (*x*)^+^ represents max{*x*, 0}. diag(**x**) denotes a diagonal matrix whose main diagonal is **x**. [**A**]_*ij*_ is the element *a*_*ij*_ of matrix **A** and **I** is the identity matrix of compatible size.

## 3. System Model

Consider a single-cell with a single base station equipped with *N*_*t*_ antennas and *K* single antenna active users competing for resources. We assume perfect CSI at the base station and the channel coefficients are modeled as independent random variables with a zero-mean circularly symmetric complex Gaussian distribution (Rayleigh fading). The signal received by the *i*th user is given by

(1) $$\begin{array}{c} y_{i}=h_{i}x+n_{i}, \end{array}$$

where **x** ∈ *ℂ* ^*N*_*t*_×1^ is the transmitted signal vector from the base station antennas and **h**_*i*_ ∈ *ℂ* ^1×*N*_*t*_^ is the channel vector to the user *i*. Each user treats the signals intended for other users as interference and *n*_*i*_ ~ *𝒞𝒩*(0, *σ*_*n*_^2^) is the additive zero-mean white Gaussian noise with variance *σ*_*n*_^2^. The entries of the block fading channel **H** = [**h**_1_^*H*^,…, **h**_*K*_^*H*^]^*H*^ and **n** = [*n*_1_,…, *n*_*K*_]^*T*^ are normalized so that they have unitary variance, and the transmitter has an average power constraint *𝔼*{**x** ^*H*^ **x**} ≤ *P*. Since the noise has unit variance, *P* represents the total transmit signal-to-noise-ratio (SNR).

For linear spatial processing at the transmitter, the beamforming matrix can be defined as **W** = [**w**_1_, **w**_2_,…, **w**_*K*_], the symbol vector as **s** = [*s*_1_, *s*_2_,…, *s*_*K*_]^*T*^, and **P** = diag(*p*_1_,…, *p*_*K*_) is the power loading, so that the transmitted signal is given by $x=\sum_{k=1}^{K}\sqrt{p_{k}}w_{k}s_{k}$. The signal-to-interference-plus-noise ratio (SINR) of the *i*th user is

(2) $$\begin{array}{c} \text{SIN}{\text{R}}_{i}=\frac{p_{i}{\left|h_{i}w_{i}\right|}^{2}}{\sum_{j\neq i}p_{j}{\left|h_{i}w_{j}\right|}^{2}+\sigma_{n}^{2}}. \end{array}$$

Assuming *N*_*t*_ ≥ *K*, the sum rate maximization problem using beamforming (BF) can be formulated as

(3) RBF=maxW,P∑k=1Klog2(1+SINRk)  subject to  ||WP||F2≤P.

### 3.1. Zero-Forcing Beamforming

In ZFBF the channel matrix **H** at the transmitter is processed so that orthogonal channels between the transmitter and the receiver are created, defining a set of parallel subchannels. Assuming *K* active users, then for the case where *K* ≤ *N*_*t*_ and rank(*H*) ≤ *N*_*t*_, the ZF beamforming matrix is given by the Moore-Penrose pseudoinverse of **H** [17, 18] as

(4) $$\begin{array}{c} W=H^{†}=H^{H}{\left(HH^{H}\right)}^{-1}. \end{array}$$

The throughput when ZFBF is applied to (3) is given by [4]:

(5) $$\begin{array}{c} R^{\text{ZFBF}}\left(H\right)=\overset{K}{\underset{i=1}{\sum }}{\left(\log\left(\mu b_{i}\right)\right)}^{+}, \end{array}$$

where *b*_*i*_ = {[(**H** **H** ^*H*^)^−1^]_*ii*_}^−1^ is the effective channel gain of the *i*th user and its allocated power is

(6) $$\begin{array}{c} p_{i}={\left(\mu b_{i}-1\right)}^{+}, \end{array}$$

and the water level *μ* is chosen to satisfy

(7) $$\begin{array}{c} \underset{i\in \Omega }{\sum }{\left(\mu -\frac{1}{b_{i}}\right)}^{+}=P. \end{array}$$

### 3.2. Zero-Forcing Dirty Paper Beamforming

Suboptimal throughput maximization in Gaussian BC channels has been proposed in several works [4, 9, 10] based on the QR-type decomposition [18] of the channel matrix **H** = **L** **Q** obtained by applying Gram-Schmidt orthogonalization to the rows of **H**. **L** is a lower triangular matrix and **Q** has orthonormal rows. The beamforming matrix given by **W** = **Q** ^*H*^ generates a set of interference channels:

(8) $$\begin{array}{c} y_{i}=l_{ii}\sqrt{p_{i}}s_{i}+\underset{j<i}{\sum }l_{ij}\sqrt{p_{j}}s_{j}+n_{i},\;i=1,\ldots ,k, \end{array}$$

while no information is sent to users *k* + 1,…, *K*. In order to eliminate the interference component $I_{i}=\sum_{j<i}l_{ij}\sqrt{p_{j}}s_{j}$ of the *i*th user, the signals $\sqrt{p_{i}}s_{i}$ for *i* = 1,…, *k* are obtained by successive dirty-paper encoding, where *I*_*i*_ is noncausally known. This precoding scheme was proposed in [4] and the authors showed that the precoding matrix forces to zero the interference caused by users *j* > *i* on each user *i*; therefore this scheme is called zero-forcing dirty-paper (ZFDP) coding. The throughput achieved in (3) under the ZFDP scheme is given by [4]

(9) $$\begin{array}{c} R^{\text{ZFDP}}\left(H\right)=\overset{K}{\underset{i=1}{\sum }}{\left(\log\left(\mu d_{i}\right)\right)}^{+}, \end{array}$$

where *d*_*i*_ = |*l*_*ii*_|^2^ and *μ* is the solution to the water-filling equation

(10) $$\begin{array}{c} \underset{i\in \Omega }{\sum }{\left(\mu -\frac{1}{d_{i}}\right)}^{+}=P, \end{array}$$

which defines the *i*th power as *p*_*i*_ = (*μd*_*i*_ − 1)^+^.

## 4. The User Selection Problem

Let *Ω* = {1,…, *K*} be the set of all competing users where *K* is larger than the number of available antennas at the base station; that is, |*Ω* | = *K* > *N*_*t*_. Under this condition, user selection is required and the joint sum rate maximization (3) and user selection problem can be defined as

(11) $$\begin{array}{c} ℜ=\underset{𝒮\subset \Omega :|𝒮|=N_{t}}{\max}R^{\left(\text{type}\right)}\left(H\left(𝒮\right)\right), \end{array}$$

where *𝒮* ⊂ *Ω*, **H**(*𝒮*) is a row-reduced channel matrix containing only the channel vectors of the selected users and type denotes the precoder that is used, either ZFBF or ZFDP. Observing that in (11) the set of selected users is constrained to have maximum cardinality, full spatial multiplexing is sought. For the high SNR regime and ZFBF using water-filling based power allocation it is possible to achieve a final subset with cardinality *N*_*t*_ as long as the given SNR is above a critical value [4].

The optimum solution to (11) requires an exhaustive search over a search space of size $\left(\begin{array}{c} K\\ N_{t} \end{array}\right)$ and for large values of *K* its computation has prohibitive complexity. Therefore, low-complexity suboptimal algorithms have been proposed in the literature in order to maximize the throughput solving (11) in two phases (class-B approach): first by finding a set *𝒮* of quasiorthogonal users (combinatorial search) and second by allocating resources to such a set (convex optimization) [5, 6, 8].

### 4.1. Metric of Orthogonality

In the literature of user selection for MIMO systems [5, 9, 10], one of the most common approaches to form the set of selected users *𝒮* is to find iteratively the user that locally maximizes the sum power projection. This means that given *𝒮* ≠ *∅*, the optimum new user form *Ω* achieves the largest amount of projection power once its channel is projected onto the subspace spanned by the previously selected users Sp(**H**(*𝒮*)).

This procedure is optimum when only 1 element from *Ω* must be selected to be added to *𝒮*. In the case of |*𝒮* | <*N*_*t*_ the aggregation of a new user is required to meet the constraint of (11) and the aforementioned procedure results in a suboptimal maximization of the total sum of projection powers.

Let **Q**_*𝒮*_ be the orthogonal complement projector matrix of Sp(**H**(*𝒮*)) defined as [17]

(12) $$\begin{array}{c} Q_{𝒮}=I_{N_{t}}-P_{𝒮}=I_{N_{t}}-H{\left(𝒮\right)}^{H}{\left(H\left(𝒮\right)H{\left(𝒮\right)}^{H}\right)}^{-1}H\left(𝒮\right), \end{array}$$

where **P**_*𝒮*_ is the orthogonal projector matrix of Sp(**H**(*𝒮*)). In [9] Tu and Blum proposed a greedy algorithm originally designed to be applied to ZFDP coding scheme, which selects *N*_*t*_ out of *K* rows of the channel matrix **H**. Such user selection methodology is based on an iterative null space projection (NSP) and it achieves the best suboptimal solution to the problem (11) for a class-B algorithm regardless of the coding scheme, which will be elaborated upon in the following sections. In [9] given *𝒮* ≠ *∅* the new selected user is the one that maximizes the following metric:

(13) $$\begin{array}{c} r_{𝒮,i}=h_{i}Q_{𝒮}h_{i}^{H}=h_{i}h_{i}^{H}-h_{i}P_{𝒮}h_{i}^{H}, \end{array}$$

where the term **h**_*i*_ **P**_*𝒮*_ **h**_*i*_^*H*^ represents the power loss due to the imperfect orthogonality between **h**_*i*_ and Sp(**H**(*𝒮*)). In other words, the metric *r*_*𝒮*,*i*_ measures the amount of power preserved by user *i* when **h**_*i*_ is projected onto the null space of **H**(*𝒮*). The same idea of [9] has been applied by Wang and Yeh [8] for ZFBF calculating the null space of **H**(*𝒮*) via SVD.

**Figure 1:**
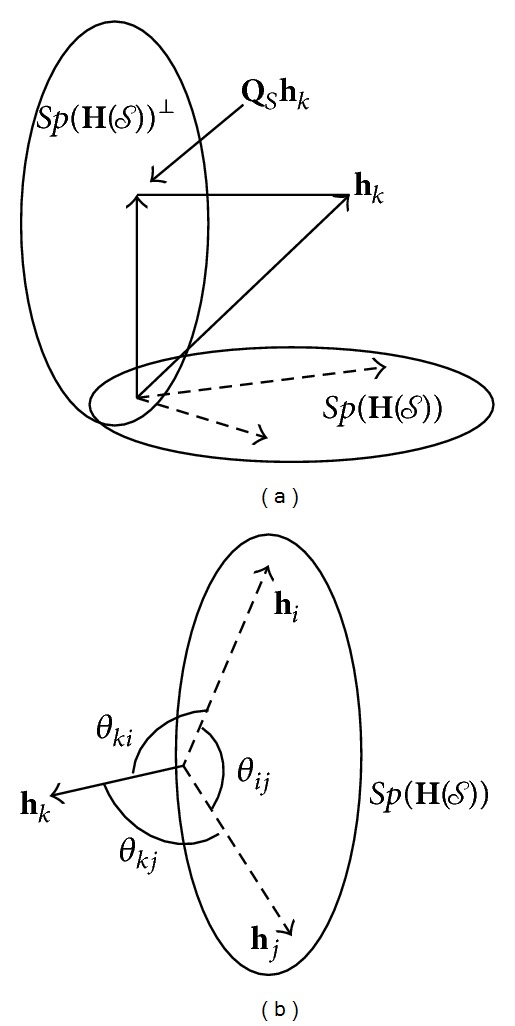
(a) The orthogonal component of vector **h**_*k*_ to Sp(**H**(*𝒮*)). (b) Physical components of the interaction of two selected users *i* and *j* with third unselected user *k*.

This concept is represented in Figure 1(a) where the channel **h**_*k*_ of the *k*th unselected user is projected onto the null space Sp(**H**(*𝒮*))^⊥^ using (12).

Several user selection algorithms (e.g., [5, 6, 11, 16, 19, 20]) attempt to create groups of quasiorthogonal users based on the information provided by the coefficient of correlation *η*_*ij*_ which for two users *i* and *j* is defined as [17, 21]

(14) $$\begin{array}{c} \eta_{ij}=\cos\left(\theta_{ij}\right)=\frac{\langle h_{i},h_{j}\rangle }{||h_{i}||||h_{j}||},\;0\leq \theta_{ij}\leq \pi , \end{array}$$

where the coefficient 0 ≤ |*η*_*ij*_ | ≤1 geometrically represents the cosine of the angle between the two channel vectors [17]. In [22] the authors presented an algorithm that selects the best 2 users out of *K*. The first user *i* ∈ *𝒮* is given by the user with the highest channel norm as in [5, 6, 8, 9], and the second user *j* ∈ *Ω* is the one that maximizes the product ||**h**_*j*_||^2^(1 − *η*_*ij*_^2^) = ||**h**_*j*_||^2^sin^2^(*θ*_*ij*_). In the particular case of [22] when |*𝒮* | = 1, **h**_*j*_ **Q**_*𝒮*_ **h**_*j*_^*H*^ = ||**h**_*j*_||^2^sin^2^(*θ*_*ij*_); that is, scaling the squared norm by the squared sine of the angle between user *i* and *j* is equivalent to projecting **h**_*j*_ onto the null space of **h**_*i*_ [17]. When zero-forcing-based precoding is used, the term sin^2^(*θ*_*ij*_) can be viewed as a projection power loss factor [21]. In the following section we derive a metric to approximate the projection of a given **h**_*i*_, ∀*i* ∉ *𝒮* onto Sp(**H**(*𝒮*))^⊥^ for the general case where |*𝒮* | >1.

## 5. Power Projection Based User Selection

In this section we propose a cross-layer design that suboptimally solves the sum rate maximization problem. This design only considers the physical layer model and we ignore the application level delay effects and assume that all users have infinite information to transmit when they are scheduled. The generalization of the user selection problem is modeled as an integer convex program and we analyze the suboptimality of the selection metrics.

### 5.1. Iterative Power Projection (IPP) Algorithm

Based on the fact that (13) has a fundamental connection to the coefficients of correlation, we design an algorithm that attempts to find a quasiorthogonal set of users *𝒮* using exclusively the information provided by the channel norms and the orthogonality between any two user channels given by (14).  Figure 1(b) exemplifies the required information used to find the set *𝒮*, and for two selected users *i* and *j* the figure shows the physical components that affect the interaction with a third unselected user *k*.

In order to start the users selection process, we assume that the base station knows the coefficients of correlation for all users in *Ω* = {1,…, *K*}, which requires (*K* ^2^ − *K*)/2 computations of (14) since *η*_*ij*_ = *η*_*ji*_ and the computation of the coefficients (inner product and vector norm operations) can be done within time *𝒪*(*K*). For the sake of notation let *ϱ*_*ij*_ = 1 − *η*_*ij*_^2^, ${\hat{\varrho }}_{ij}=1-|\eta_{ij}|$ and define the following geometric and arithmetic means for the elements *ϱ* associated with user *i* ∈ *Ω* as:

(15) $$\begin{array}{c} M_{g(i)}={\left(\underset{j\neq i,j\in \Omega }{\prod }\varrho_{ij}\right)}^{1/\left(|\Omega |-1\right)}\leq \left(\frac{1}{|\Omega |-1}\right)\underset{j\neq i,j\in \Omega }{\sum }\varrho_{ij}, \end{array}$$

where *M*_*g*(*i*)_ is a lower bound of the arithmetic mean of the projection power loss factors of user *i*. We select the first user as the one that preserves the highest amount of average power once it is projected onto all other users, such that

(16) $$\begin{array}{c} i^{*}=\arg\underset{i\in \Omega }{\max}{||h_{i}||}^{2}M_{g(i)}, \end{array}$$

and the sets of selected and unselected users are updated, *𝒮* = {i*} and *Ω* = *Ω* − {i*}. By selecting the first user using (16) the goal is assigning priority weights to the channel norms; that is, users with large channel norms are penalized if their associated correlation coefficients have a large variance. Furthermore, the geometric mean *M*_*g*(*i*)_ minimizes the bias created by the terms *ϱ* with very large or small values, which would be neglected if the arithmetic mean of the projection power loss factors were considered in (16).

The following user to be selected must maximize two criteria at the same time. On the one hand, it must maximize its own projected power which is affected by the coefficients *ϱ* of the already selected users in *𝒮*. The effective projected power of the user *i* ∈ *Ω* is given by

(17) $$\begin{array}{c} \psi_{i}={||h_{i}||}^{2}\underset{j\in 𝒮}{\prod }\varrho_{ij}. \end{array}$$

On the other hand, the users in *𝒮* have already achieved an effective projected power that is defined as:

(18) $$\begin{array}{c} \phi_{j}={||h_{j}||}^{2}\underset{k\neq j,k\in 𝒮}{\prod }\varrho_{jk},\;j\in 𝒮. \end{array}$$

For a new user candidate *i* ∈ *Ω*, its aggregation to the set *𝒮* implies a reduction of the total sum of projected powers of the selected users (∑_*j*∈*𝒮*_ *ϕ*_*j*_) by the factors *ϱ* associated with the new selected user. Using the arithmetic and geometric means, lower bounds of the average projected power of the selected users in (18) can be defined for the *i*th unselected user as follows:

(19) $$\begin{array}{c} \underset{j\in 𝒮}{\prod }\phi_{j}\varrho_{ij}\leq {\left(\frac{1}{\left|𝒮\right|}\underset{j\in 𝒮}{\sum }\phi_{j}\varrho_{ij}\right)}^{\left|𝒮\right|}\leq {\left(\frac{1}{\left|𝒮\right|}\underset{j\in 𝒮}{\sum }\phi_{j}\right)}^{\left|𝒮\right|}. \end{array}$$

The total effective projection power ${\dot{\varphi }}_{i}$ of the unselected user *i* takes into account both the average projection power over the elements in *𝒮* computed for the lower bound in (19) and the projection power of user *i* ∈ *Ω* (17). Consider

(20) φ˙i=(∏j∈𝒮ϕjϱij)︸gain ∀j∈𝒮(||hi||2∏j∈𝒮ϱij)︸gain ∀i∈Ω=(∏j∈𝒮ϕj)︸constant ∀i∈Ω(∏j∈𝒮ϱij)(||hi||2∏j∈𝒮ϱij).

By taking the square of the product of the terms *ϱ*_*ij*_, both effects are considered: the impact of the selected users over user *i* and the power degradation that the users in *𝒮* will have if user *i* is selected.

Since the effective projected power of the selected users remains constant for all users in *Ω*, the metric in (20) can be normalized as follows:

(21) $$\begin{array}{c} \varphi_{i}={||h_{i}||}^{2}\underset{j\in 𝒮}{\prod }\varrho_{ij}^{2}. \end{array}$$

Given *𝒮*, the next selected user is found using the metric defined in (21) as

(22) $$\begin{array}{c} i^{*}=\arg\;\underset{i\in \Omega }{\max}\;\varphi_{i}, \end{array}$$

where the selection of the locally optimum *φ*(*n*) in a given iteration *n* is conditioned on the choice of *φ*(1),…, *φ*(*n* − 1).

As *K* → *∞* the number of total operations to solve problem (11) becomes computationally costly and a more efficient update of the set *Ω* can be performed. By selecting a new user using (22), each iteration requires the comparison of |*Ω*| elements in order to select the user whose projection power is maximum. Considering that the cardinality of the final set must be *N*_*t*_, without modifying *Ω*, this algorithm would require a total of *L* = *N*_*t*_(*K* − (*N*_*t*_ − 1)/2) comparison operations. For our case, the projection power evaluations for the metric used in (21) will use all coefficients *ϱ* associated with the elements of *𝒮*. The algorithms proposed in [6, 8, 9] also require *L* comparison operations versus the elements of *𝒮*. However, the computational complexity is quite different since each comparison requires a matrix multiplication, whilst the metric used in (21) is a multiplication of real positive numbers.

In [5, 20, 22] after a new user *i* is added to *𝒮*, the set of unselected user *Ω* is reduced by keeping the users whose correlation factors are above a threshold *α*_th_; that is, *Ω*(*n*) = {*j* ∈ *Ω*(*n* − 1) : *η*_*ij*_ < *α*_th_}, where *n* stands for the iteration number and *i* is the selected user of iteration *n* − 1. This subselection within the algorithm has the drawback that the value of the parameter *α*_th_ is fixed which might result in a drastic reduction of the size of *Ω* and the degradation of the multiuser diversity. According to [5] there exists an optimum value of the threshold *α*_th_ for each value of *K* and *N*_*t*_, but the mathematical relationship between these terms is not given in a closed form. The statistical dependence of the average throughput due to *α*_th_ has been established only for the case where the cardinality of the set of selected users is constrained to be 2; that is, |*𝒮* | = 2 in [21].

We propose a dynamic reduction of the set *Ω* considering two factors to discard users at each iteration. The first criterion is related to the statistics of the projection powers regarding the users that have been selected. The second criterion weights the first criterion based on the number of active users and the number of antennas *N*_*t*_. Let us define the arithmetic mean of the projected powers given the new selected user i* as

(23) $$\begin{array}{c} M_{a(i^{*})}=\frac{1}{\left|\Omega \right|}\underset{j\in \Omega }{\sum }{||h_{j}||}^{2}\varrho_{i^{*}j}. \end{array}$$

Notice that the power projection computation is performed considering only the power projection loss factors associated with i*, and each term of the sum in (23) is the multiplication of two real numbers. The metric defined in (23) is used to discard users whose projection powers are below the arithmetic mean which results in a reduction of the number of comparisons for the next iteration. Nevertheless, when the number of total users is low (*K* ≈ *N*_*t*_) the number of users in *Ω* should not be reduced drastically in order to preserve enough multiuser diversity and to achieve full spatial multiplexing. We define a weight factor based on the number of antennas *N*_*t*_ and the size of the sets *𝒮* and *Ω* as follows:

(24) $$\begin{array}{c} w_{\left(N_{t},𝒮,\Omega \right)}=1-{\left(\frac{N_{t}-|𝒮|}{\left|\Omega \right|}\right)}^{1/\left(N_{t}-|𝒮|\right)}. \end{array}$$

The objective of *w*_(*N*_*t*_,*𝒮*,*Ω*)_ is to scale *M*_*a*(i*)_ in iteration *n* taking into account the degrees of freedom available at the base station (rank(**H**(*𝒮*))) and the current size of *Ω*. Given the new selected user i* and weighting (23) by (24), the modified set of users that will compete to be scheduled in the next iteration *n* + 1 is defined as

(25) $$\begin{array}{c} \Omega \left(n+1\right)=\left\{j\in \Omega \left(n\right):{||h_{j}||}^{2}\varrho_{i^{*}j}\geq w_{\left(N_{t},𝒮,\Omega \right)}M_{a(i^{*})}\right\}. \end{array}$$

The procedure to generate the quasiorthogonal set of user that solves problem (11) is described in Algorithm 1.

### 5.2. User Selection as an Integer Linear Program (ILP)

The optimization performed in Algorithm 1 can be described as a greedy search over a tree structure [23] where the tree's root is given by the element of *Ω* that preservers a higher average projected power (16). Similar approaches are implemented in [5, 6, 8, 9] considering the user with the maximum channel norm as the root of tree. The greedy Algorithm 1 makes a sequence of decisions in order to optimize the metric in (22). However, this local optimization might not lead to a global optimal solution. Moreover, since the first user is found by (16), the correlation of such a user with the future selected users is neglected when *𝒮* is initialized. A general mathematical model of the interaction of all elements in *𝒮* that exploits the metrics used in (16) and (22) can be designed. Due to the structure of (16) and (22) which maximizes the squared channel norm weighted by the product (interaction) of the correlation coefficients, we can model a relaxed version of the user selection problem (11) as an integer programming problem.

Let us define the interaction of the user *i* ∈ *Ω* with the rest of the users as a function *f*_*i*_ considering the structure of (21) as

(26) $$\begin{array}{c} f_{i}={||h_{i}||}^{2}\underset{j\neq i}{\prod }\varrho_{ij}^{2},\;\forall i,j\in \Omega , \end{array}$$

and by applying a change of variables, the function ${\tilde{f}}_{i}=\log(f_{i})$ is given by

(27) $$\begin{array}{c} {\tilde{f}}_{i}=a_{i}+\underset{j\neq i}{\sum }b_{ij}, \end{array}$$

where *a*_*i*_ = 2log(||**h**_*i*_||) and *b*_*ij*_ = 2log(*ϱ*_*ij*_). Our objective is to maximize the total sum of the projected powers which is a function of two factors, the orthogonality between the selected channels and the amount of remaining power after a projection. Therefore, (11) can be thought of as the maximization of $\sum_{i}{\tilde{f}}_{i}$ with the constraint that |*𝒮* | = *N*_*t*_. In order to introduce such constraint, we define the following binary variable *y*_*i*_ as

(28) $$\begin{array}{c} y_{i}=\{\begin{array}{cc} 1 & \text{if user}\;\;i\;\;\text{is selected}\\ 0 & \text{otherwise.} \end{array} \end{array}$$

In the same way we can define a set of binary variables *x*_*ij*_ that relate to the common coefficient *ϱ*_*ij*_ of two users as

(29) $$\begin{array}{c} x_{ij}=\{\begin{array}{cc} 1 & \text{if both users}\;\;i\;\;\text{and}\;\;j\;\;\text{are selected}\\ 0 & \text{otherwise.} \end{array} \end{array}$$

The mathematical model for the user selection problem based exclusively on the channel norms and correlation coefficients is given by

(30) maximize ∑iaiyi+2∑i∑j=i+1bijxijsubject to ∑iyi=Nt      yi+yj≤1+xij, ∀i,j      xij≤yi, ∀i,j      xij≤yj, ∀i,j      yi∈{0,1}, ∀i      xij∈{0,1}, ∀i,jvariables  yi,xij,

where (30) is a binary programming problem that generalizes the objective function optimized by Algorithm 1. The advantage of this formulation is that the order in which the users are selected has no impact on the orthogonality of the elements of **H**(*𝒮*); that is, the negative effects of selecting local optimum users in each iteration are canceled. The solution to the user selection problem is given by the binary variables *y*_*i*_ and power allocation based on water-filling is performed over the set of selected users according to the employed precoding scheme. Observe that a conversion from ${\tilde{f}}_{i}$ to *f*_*i*_ is not required, because the relevant information to form the set *𝒮* is given by the variables *y*_*i*_ that have achieved a value of one. Since the objective function is convex and the constraints are given by affine functions, this problem can be solved by the pseudodual simplex method [24] for integer programs or by using standard optimization packages [25, 26]. Moreover, problem (30) always has a feasible solution because the only constraint that might lead to infeasibility is the equality constraint that is always met due to the fact that *K* ≥ *N*_*t*_. Problem (30) is a relaxed version of (11) and it finds a suboptimal solution to the user selection problem owing to the nature of the coefficients *b*_*ij*_ which is analyzed in the following subsection.

### 5.3. Suboptimality of the User Selection Process

The projection power found by (13) has a direct relationship with the correlation coefficients *η* of the users in *𝒮* and the channel vector **h** of the candidate user in *Ω*. The normalized power loss of such user once it is projected onto **P**_*𝒮*_ is called the coefficient of determination and is given by [17]

(31) $$\begin{array}{c} R_{𝒮,h}^{2}=\frac{hP_{𝒮}h^{H}}{hh^{H}}, \end{array}$$

where *R*_*𝒮*,**h**_^2^ measures how much the vector **h** can be predicted (correlated) from the selected vectors of **H**(*𝒮*). Notice that from (13) and (31) the projection of **h** onto the null space of Sp(**H**(*𝒮*)) is equivalent to 1 − *R*_*𝒮*,**h**_^2^ which can be evaluated from the correlation coefficients *η* as follows [17]:

(32) 1−R𝒮,h2 =(1−ηhπ(1)2)(1−ηhπ(2) ∣ π(1)2)⋯(1−ηhπ(k) ∣ π(1)⋯π(k−1)2),

where *π*(*i*) is the *i*th ordered element of **H**(*𝒮*) and *η*_**h***π*(*k*)∣*π*(1)⋯*π*(*k*−1)_ is the partial correlation between the candidate vector **h** and the ordered channel vector **h**_*π*(*k*)_ ∈ **H**(*𝒮*) associated with *π*(*k*) eliminating the effects due to *π*(1), *π*(2),…, *π*(*k* − 1). The exact computation of the last *k* − 1 partial correlation coefficients in (32) requires the implementation of recursive algorithms whose analysis and efficient implementation are a subject of future research. It can be observed that the product that scales the squared channel norm of user *i* in (21) contains all the information of the correlation coefficients of elements of *𝒮* which resembles the product (32). However, (21) considers redundant information of how all elements in **H**(*𝒮*) interact with **h** which results in a suboptimal evaluation of (32). Notice that as *K* grows, the probability that basis of Sp(**H**(*𝒮*)) can describe a new candidate user's channel **h** decreases. Therefore, the gap between the correlation and the partial correlation factors reduces as well. This characteristic is used in [6] to prove that for *K* → *∞* the performance of an SVD-based scheduling algorithm that generates a quasiorthogonal set of users by approximating (31) achieves asymptotical optimal user selection performance.

The optimum metric for user selection varies according to the precoding scheme that is implemented. For the case of ZFDP, the fact that (21) considers redundant information when all terms *ϱ* are multiplied can be compensated by the elimination of the noncausally known interference. In the case of ZFBF the orthogonality among selected channels plays a more important role in terms of throughput maximization. In order to compensate the lack of knowledge of the partial correlation coefficients in (32), we consider larger values of the power loss factors; that is, the procedure for user selection is the one described in Algorithm 1 with the difference that for the ZFBF scheme we use ${\hat{\varrho }}_{ij}$ instead of *ϱ*_*ij*_. Due to the fact that ${\hat{\varrho }}_{ij}\leq \varrho_{ij}$ (with equality when the channels are uncorrelated) the projection power loss factor increases its value, and in this way the poor orthogonality between channels has a higher impact when the squared channel norms are scaled in (21).

## 6. Numerical Results

We compare the proposed user selection algorithm with several state-of-the-art algorithms, namely the semiorthogonal user selection (SUS) proposed in [5] with threshold parameter *α*_th_ and the null space projection based approach (NSP) [8, 9]. The upper bound of the sum rate is given by the expected value of the solution of (11) found by an exhaustive search. In order to highlight the contribution of multiuser diversity we compare performance with respect to two simplistic user selection approaches, one based on the maximum channel gain (MCG) criterion (selecting the *N*_*t*_ users with higher channels norms), and a second approach performing round robin user scheduling (RRS) policy. We also compare the performance of the proposed Algorithm 1 (IPP) with two greedy class-A algorithms, one proposed by Dimić and Sidiropoulos [10], and the other proposed by Karachontzitis and Toumpakaris [11]. The solution of the integer linear program (ILP) optimization in (30) is presented and used as an upper bound of the performance of Algorithm 1 (IPP) and compared to the optimum solution of (11). The simulations consider perfect CSIT; fading channels are generated following a complex Gaussian distribution with unit variance and the average sum rate is given in [bps/Hz]. Since we evaluate system performance via Shannon capacity by means of (5) and (9), the results are independent of the specific implementation on the coding and modulation schemes, which provides us with a general design insight.

### 6.1. Throughput (*R*) versus Number of Active Users (*K*)

**Figure 2:**
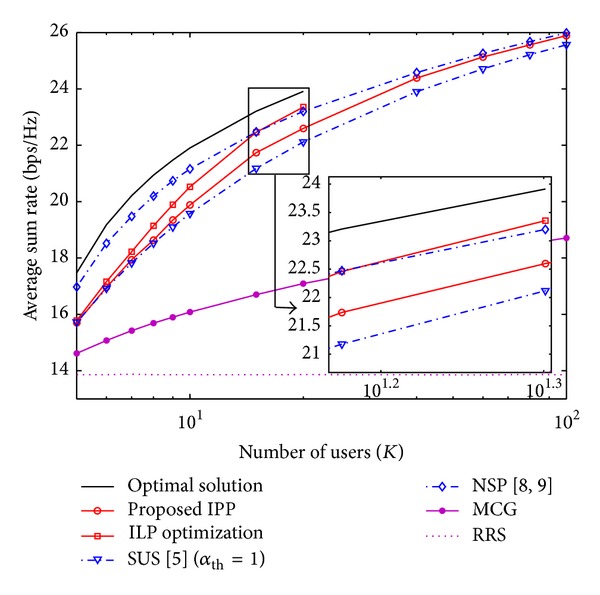
Average sum rate as a function of the number of users *K* for the ZFBF scheme with SNR = 18 [dB] and *N*_*t*_ = 4.

**Figure 3:**
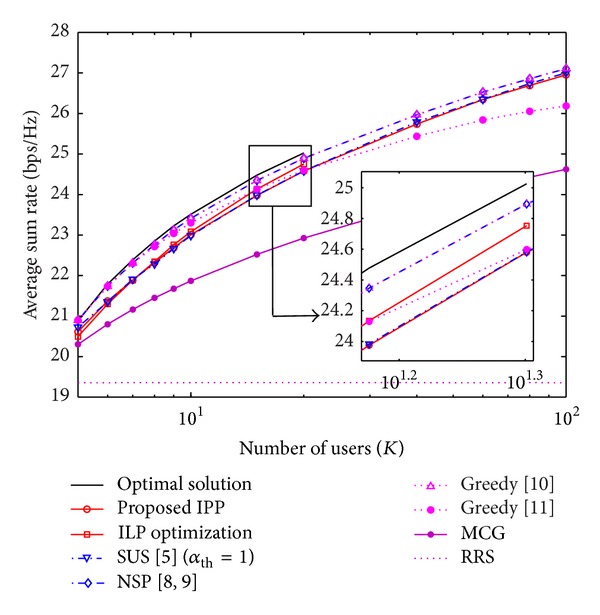
Average sum rate as a function of the number of users *K* for the ZFDP scheme with SNR = 18 [dB] and *N*_*t*_ = 4.

In Figures 2 and 3, we compare the throughput performance of different user selection strategies and Algorithm 1 regarding the number of competing users *K*. The performance of ZFBF is highly susceptible to the characteristics of the set of selected users *𝒮*. IPP algorithm performs the user selection exploiting the information of the terms $\hat{\varrho }$. Since ${\hat{\varrho }}_{ij}\leq \varrho_{ij}$, the consequence is a more drastic reduction in the power projection in (21) due to the value of the correlation coefficient *η*_*ij*_.  Figure 2 shows that IPP achieves a considerable portion of the average sum rate of the optimum selection; in the case when *K* = 5 the performance gap regarding the optimum user selection is about 11%. For *K* = 10, IPP achieves 90% of the optimum users selection's sum rate and outperforms SUS (*α*_th_ = 1). It is worth mentioning that the parameter *α*_th_ has the function of dropping users whose correlation factor is below its value as described in Subsection 5.1. In this case we select *α*_th_ = 1 in order to guarantee that the set constraint in (11) is not violated. The objective of IPP algorithm is to achieve the performance of the greedy user selection based on the null space projection (NSP). The performance of the IPP algorithm has an asymptotic behavior regarding the NSP approach as *K* grows. For *K* = 20, IPP achieves roughly 97% of the sum rate of the NSP based algorithms [8, 9].

A comparison of the IPP algorithm to the ILP optimization shows that the latter exploits more efficiently the user diversity as *K* grows. It is interesting that for *K* ≥ 20 the ILP optimization achieves better performance than the NSP approach in Figure 2. This result suggests that there exists a critical value of *K* for which the user selection of the ILP optimization overcomes the selection performed using the metric defined in (13). For *K* = 20, the performance gap between the optimum user selection and the ILP optimization is less than 5%. This means that for given deployment *N*_*t*_, there exists a finite value *K*_0_ for which ∀*K* > *K*_0_ the sum rate gap between the exhaustive search and the model (30) is negligible. However, the complexity of computing the solution of (30) grows exponentially with *K* which is impractical (infeasible) for online implementations, but it is still an appealing approximation to (11) compared to the large search space size of the optimum solution for moderate values of *K*.

The performance of the IPP is determined by the precoding scheme that is used. For ZFDP in Figure 3, it can be observed that IPP performs as well as SUS but there is still a performance gap compared to the NSP approach. For *K* = 20, IPP achieves the same performance of the greedy selection of [11] and 98% and 99% of the sum rate of the optimum selection and the NSP approach, respectively. For ZFDP and *K* ≥ 8, the ILP optimization achieves better performance than IPP but is not effective enough to reach the performance of the NSP approach for low values of *K*. Nevertheless, for *K* = 20, the ILP optimization achieves 98% of the sum rate of the optimum selection. IPP shows an asymptotic performance as *K* → *∞* with respect to the NSP approach and the optimum selection for both precoding schemes.

### 6.2. Throughput (*R*) versus SNR (*P*)

**Figure 4:**
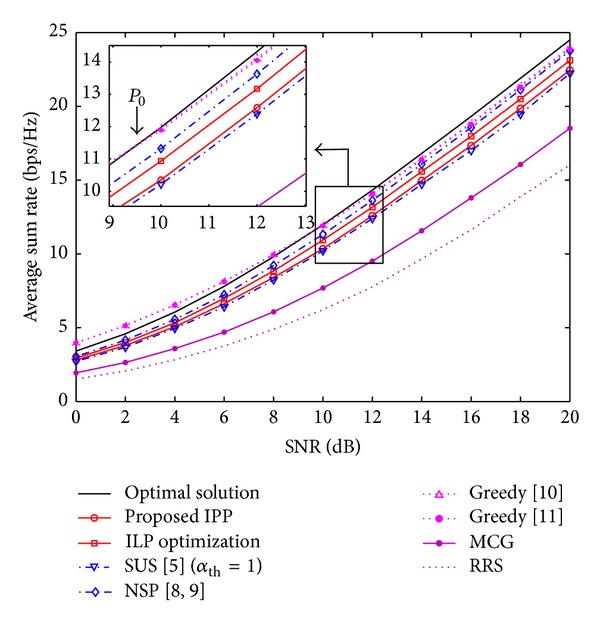
Average sum rate as a function of the SNR for ZFBF scheme with *K* = 10 and *N*_*t*_ = 4.

**Figure 5:**
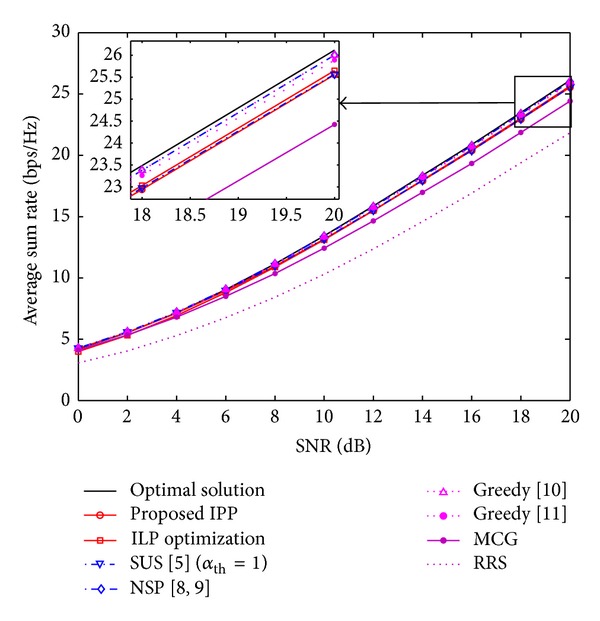
Average sum rate as a function of the SNR for ZFDP scheme with *K* = 10 and *N*_*t*_ = 4.

For zero-forcing-based beamforming, we know from [4] that for a given SNR (*P*) the maximum throughput *ℜ* under the constraint |*𝒮* | ≤*N*_*t*_ in (11) might be achieved by a set of selected users of cardinality strictly less than rank(**H**(*𝒮*)). Nevertheless, from the properties of water-filling power allocation in (5), there exists a finite value *P*_0_ (which depends on **H**(*𝒮*)) for which ∀*P* ≥ *P*_0_, *ℜ* is achieved by a subset of cardinality *N*_*t*_. Notice that since the greedy class-A algorithms in [10, 11] obey the constraint |*𝒮* | ≤*N*_*t*_, the sum rate that they achieve for *P* < *P*_0_ is higher than the capacity of the optimal solution in (11) but the number of scheduled users is less. This phenomenon can be observed in Figure 4 where for a given number of user *K* = 10, the value of *P*_0_ ≈ 10 [dB] and the optimum solution of (11) are always better than the solution of the algorithms in [10, 11]. It is worthy to point out that the optimum user selection here presented is found in a search space of size $\left(\begin{array}{c} K\\ N_{t} \end{array}\right)$ in a class-B algorithm, whilst the search space in class-A algorithms [10, 11] has a size of $\sum_{n=1}^{N_{t}}\left(\begin{array}{c} K\\ n \end{array}\right)$, which has no constraints on the minimum number of selected users. Therefore, the optimum solution shown in our results is valid only for class-B algorithms and presenting class-A algorithms have as objective to highlight the difference between classes. Considering the high SNR regime (10 ≤ *P* ≤ 20) in Figure 4, the performance gap between IPP and the optimum solution ranges from 14% to 9% and for the NSP approach the performance gap goes from 9% to 4% in the same SNR range. For the case of ZFBF, the ILP optimization achieves a better approximation to NSP than the IPP approach. However, in the case of ZFDP in Figure 5, the performance gap between IPP and the ILP optimization is about 1%, and both approaches achieve roughly 98% of the optimum selection capacity for SNR of 20 dB. An interesting fact is that the MCG selection achieves 93% the optimum selection capacity for *K* = 10 and *P* = 20 dB under ZFDP. This indicates that for the high SNR regime, channel gains play a more important role for the user selection process in scenarios where nonlinear precoding can be implemented. This can result in the design of novel low-complexity user selection algorithms for specific nonlinear precoding schemes. Still, the performance of a class-B algorithm depends on the multiuser diversity and the SNR regime.

### 6.3. Cardinality of *𝒮* and *Ω*

**Figure 6:**
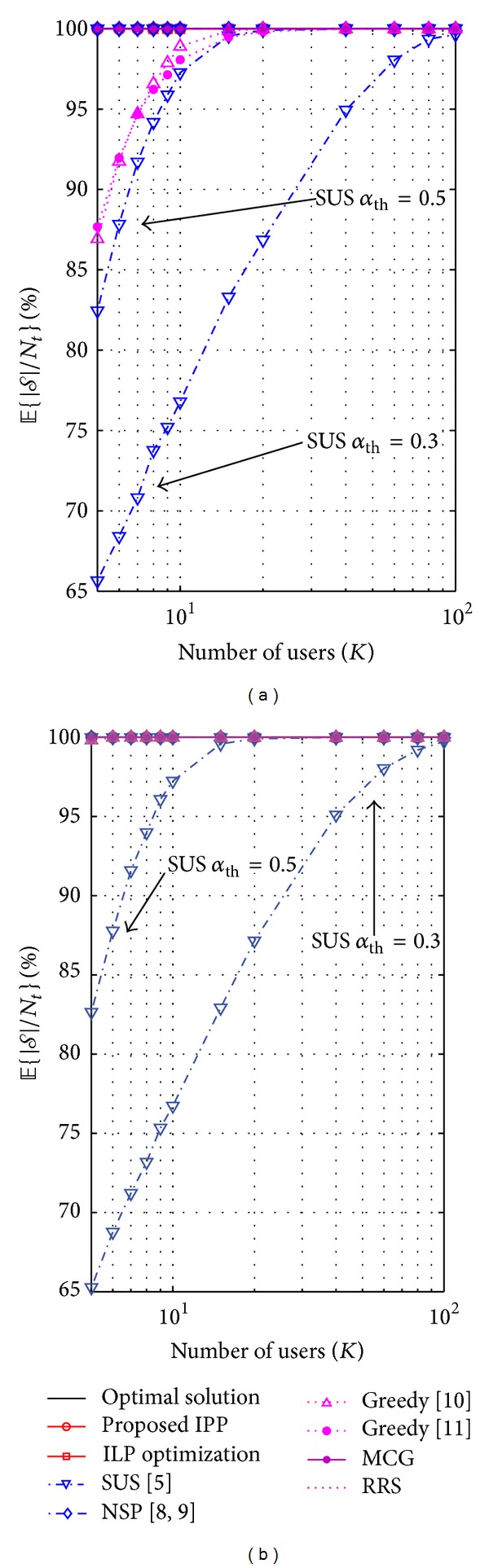
The metric *𝔼*{|*𝒮* | /*N*_*t*_} measures the degree of spatial multiplexing that is exploited for each scheduling algorithm considering SNR = 18 [dB]: (a) ZFBF and (b) ZFDP.

The cardinality of the set *𝒮* is conditioned by the class of the algorithm that is implemented, its parameters, and the type of precoding that is used. In Figure 6 we analyze in percentage the average value of the ratio |*𝒮* | /*N*_*t*_ for (a) ZFBF and (b) ZFDP. Such ratio indicates if full spatial multiplexing is achieved. In the case of ZFBF, we can see that both class-A algorithms [10, 11] require *K* ≥ 20 in order to achieve the maximum cardinality of *𝒮*. To exemplify the inconvenience of designing an algorithm dependent of nondynamic parameters, notice that setting a wrong value to the parameter *α*_th_ of the SUS algorithm might lead to a degradation of both the cardinality of the set of selected users and the sum rate. For the case of ZFDP we can see that the robustness of the precoder allows us to schedule *N*_*t*_ user in both classes of algorithms. This has a direct impact in the achieved fairness owing to the large cardinality of *𝒮*. The rate distribution among the users is improved since more users achieve a portion of the sum rate regardless of the fact that throughput maximization is the main objective of (11).

**Figure 7:**
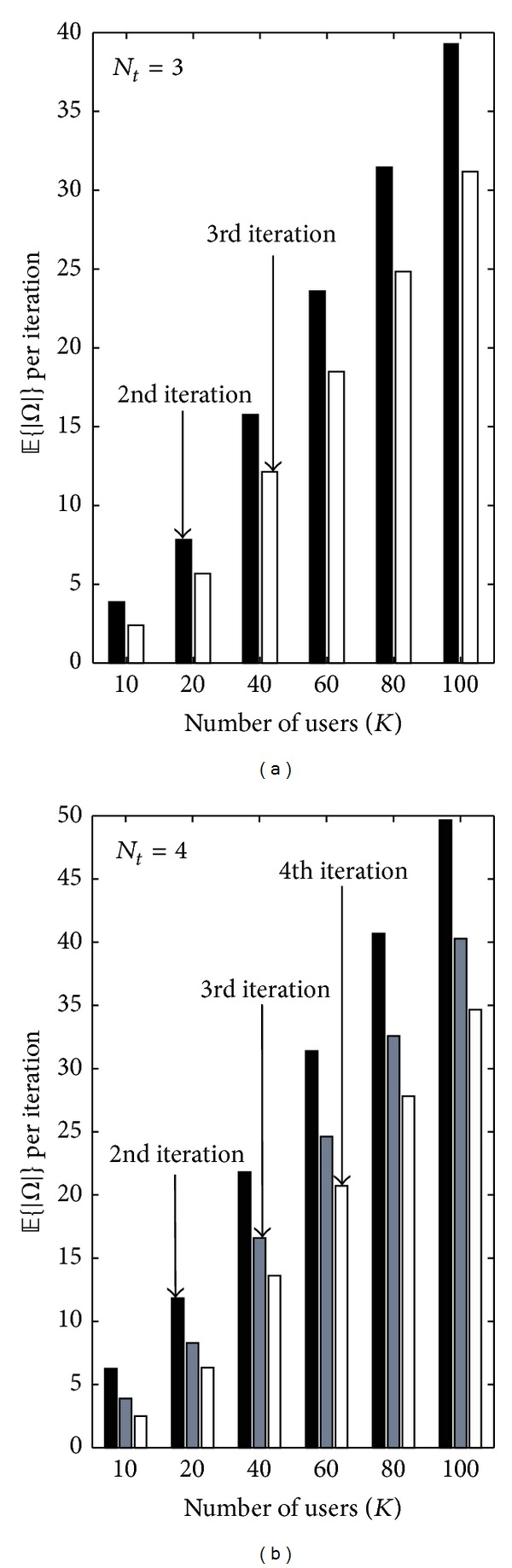
Average cardinality of the set of unselected users (*𝔼*{|*Ω*|}) for each iteration of the IPP algorithm with SNR = 18 [dB], (a) *N*_*t*_ = 3, and (b) *N*_*t*_ = 4.

With the reduction of the set *Ω* each iteration becomes relevant for high values of *K* and *N*_*t*_. The effects of (25) on the cardinality of the set of unselected users *Ω* per iterations are presented in Figure 7 for (a) *N*_*t*_ = 3 and (b) *N*_*t*_ = 4. The figures show the average number of users kept in the set *Ω* of each iteration of Algorithm 1 for different number of users. The first iteration always considers all *K* users to find the initial selected user. As the size of *𝒮* increases the number of required users to achieve |*𝒮* | = *N*_*t*_ reduces and (24) takes into account such decrement to give more or less priority to *M*_*a*(i*)_.

### 6.4. Complexity Analysis and Implementation Limitations

The complexity of solving (11) can be analyzed in two parts. The first one is the complexity required to implement each one of the precoders and the second one is the complexity of IPP. For the case of ZFBF, the precoding requires an *N*_*t*_ × *N*_*t*_ matrix inversion **W** = **H** ^†^ and for ZFDP the evaluation of the beamforming weights requires a QR-type decomposition. For both coding schemes, this process is carried out after IPP finished the user selection process. The most costly operation in IPP is the evaluation of (*K* ^2^ − *K*)/2 inner products to define the correlation coefficients that can be done in time *𝒪*(*K*). Since this values does not change along the selection process, they must be computed once and can be stored in memory. Notice that the evaluation of (16) requires a time *𝒪*(*K*) since only multiplications of real positive numbers are required and a sort operation (ordering) performed in time *𝒪*(*K* log_2_(*K*)). For the case where the set *Ω* reduces in one element per iteration and a total of *N*_*t*_ iterations are required the total complexity is *𝒪*(*KN*_*t*_ + *N*_*t*_ *K* log_2_(*K*)) ≈ *𝒪*(*KN*_*t*_). However, for the following iterations the time complexity of computing (22) is a function of the set of unselected user that is modified according the statistics of the projection power given by *M*_*a*(i*)_ and the weight *w*_(*N*_*t*_,*𝒮*,*Ω*)_. This implies that each iteration will require a time *𝒪*(|*Ω* | (1 + log_2_(|*Ω*|))) ≈ *𝒪*(|*Ω*|) and *Ω* changes for each iteration according to (25).

The solution of (30) requires the optimization over *L*_ILP_ = (1/2)*K*(*K* + 3) binary variables in the objective function. This means that a total of 2^*L*_ILP_^ configurations of those variables are available and the number of valid configurations depends on the constraints imposed over the binary variables. Regardless of the existence of pseudopolynomial algorithms that solve integer programs avoiding the evaluation of all configurations [24], real time computation of the solution of (30) is prohibited for large values of *K*.  Table 1 summarizes the time complexity of different user selection algorithms.

The proposed algorithms assume perfect CSIT. However, in practical systems it is difficult to guarantee this condition. Even if channel estimation is very accurate, there is an error in the channels at the transmitter due to mobility and feedback delays. Several works (e.g., [5, 8, 27]) showed that outdated CSIT destroys the quasiorthogonality of the selected channels which degrades the performance of zero-forcing-based transmission schemes. Orthogonality can be fully exploited when there is near to perfect CSIT. The authors in [27] showed that a significant fraction of the sum rate with perfect CSIT can be achieved if the ratio between the outdated channel at the transmitter and the estimation error is kept above a threshold. Therefore, as the frame lengths are designed so that magnitude of the real channels and the errors due to outdated estimates maintain a given average ratio, the proposed user selection techniques are effective.

## 7. Conclusions

In this paper, we presented a low-complexity algorithm that finds a quasiorthogonal set of users that maximizes the system throughput for MIMO BC channels using linear ZFBF and nonlinear ZFDP beamforming schemes. We exploited a fundamental relation between the projection power loss factors related to the correlation coefficients and the orthogonal complement projector matrix related to the null space of the selected channels. Our algorithm approximates the projected power using a metric that is based exclusively on the physical characteristics of the channels whose accuracy increases with the number of competing users. However, the dependence of the multiuser diversity is not critical and for a moderate number of users the algorithm achieves a good trade-off between performance and complexity. We compared the proposed algorithm to different state-of-the-art algorithms and numerical results show a small performance gap between the optimum user selection and the proposed algorithm. We also presented an integer program model that approximates the performance of the exhaustive search when the number of users is large and it provides an upper bound of the performance of the proposed algorithm. The results obtained by numerical simulation indicate that an efficient and low-complexity cross-layer scheduling design can profit from fundamental information that characterizes the relation between wireless channels without implementing extensive matrix operations for the user selection process.

## Figures and Tables

**Algorithm 1:**
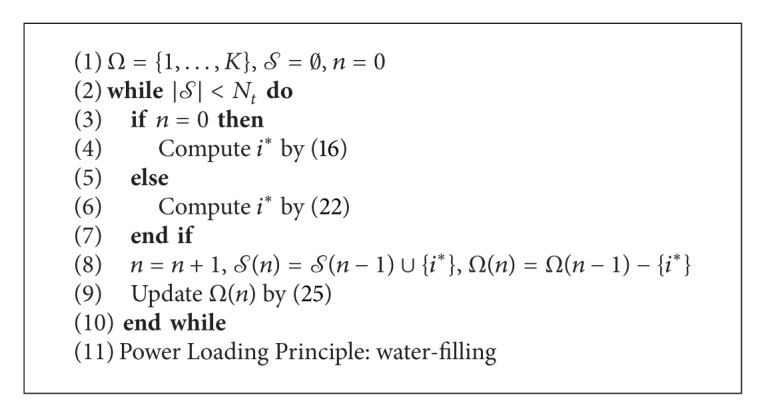
Iterative power projection (IPP).

**Table 1:** Complexity comparison of user selection algorithms.

Class A		Class B		
[10]	[11]	SUS [5]	NSP [9]	IPP
*𝒪*(*KN*_*t*_^3^)	*𝒪*(*KN*_*t*_^2^)	*𝒪*(*KN*_*t*_^3^)	*𝒪*(*KN*_*t*_^3^)	*𝒪*(*KN*_*t*_)
